# Sliding of coherent twin boundaries

**DOI:** 10.1038/s41467-017-01234-8

**Published:** 2017-10-24

**Authors:** Zhang-Jie Wang, Qing-Jie Li, Yao Li, Long-Chao Huang, Lei Lu, Ming Dao, Ju Li, Evan Ma, Subra Suresh, Zhi-Wei Shan

**Affiliations:** 1 Center for Advancing Materials Performance from the Nanoscale and Hysitron Applied Research Center in China, State Key Laboratory for Mechanical Behavior of Materials, Xi’an Jiaotong University, Xi’an, 710049 China; 2 Department of Materials Science and Engineering, Johns Hopkins University, Baltimore, MD 21218 USA; 3 Shenyang National Laboratory for Materials Science, Institute of Metal Research, Chinese Academy of Sciences, Shenyang, 110016 China; 4 Department of Materials Science and Engineering, Massachusetts Institute of Technology, Cambridge, MA 02139 USA; 5 Department of Nuclear Science and Engineering, Massachusetts Institute of Technology, Cambridge, MA 02139 USA; 6 Nanyang Technological University, 50 Nanyang Avenue, Main Campus, Singapore, 639798 Singapore

## Abstract

Coherent twin boundaries (CTBs) are internal interfaces that can play a key role in markedly enhancing the strength of metallic materials while preserving their ductility. They are known to accommodate plastic deformation primarily through their migration, while experimental evidence documenting large-scale sliding of CTBs to facilitate deformation has thus far not been reported. We show here that CTB sliding is possible whenever the loading orientation enables the Schmid factors of leading and trailing partial dislocations to be comparable to each other. This theoretical prediction is confirmed by real-time transmission electron microscope experimental observations during uniaxial deformation of copper pillars with different orientations and is further validated at the atomic scale by recourse to molecular dynamics simulations. Our findings provide mechanistic insights into the evolution of plasticity in heavily twinned face-centered cubic metals, with the potential for optimizing mechanical properties with nanoscale CTBs in material design.

## Abstract (web summary)

Coherent twin boundaries (CTBs) in face-centered cubic metals are usually considered unable to slide at room temperature. Here, the authors use in situ transmission electron microscopy and molecular dynamics to show CTB sliding in copper nanopillars when leading and trailing partial dislocations have similar Schmid factors.

## Introduction

Grain boundary sliding (GBS) occurs when two adjoining grains with different crystallographic orientations undergo a relative displacement along the boundaries^1, 2^. GBS is a plasticity mechanism whereby deformation can be accommodated even at room temperature, especially in nano-grained metals^3, 4^ that possess an average grain size typically much smaller than a hundred nanometers. However, a special type of high-angle GB, the coherent twin boundary (CTB) in face-centered cubic (FCC) metals, is generally considered to be incapable of sliding at room temperature owing to the paucity of boundary dislocations^5^. This notion has been further reinforced by the fact that no experimental observations have been reported in the literature to date documenting significant sliding of coherent twin boundaries at room temperature. Twin boundary migration in the direction perpendicular to the CTB^6–11^ or incoherent TB migration^12, 13^ in the direction parallel to the CTB, is so far the only experimentally observed mode of boundary motion involving twinned FCC metals, which is mediated by Shockley partial dislocations on consecutive (111) planes^6, 8, 12, 14–19^. Despite this lack of experimental evidence for CTB sliding (CTBS), there are existing results from computational simulations employing molecular dynamics that suggest the possibility of CTBS at relatively low temperatures when shear deformation is induced along certain crystallographic orientations of the FCC crystal (such as <110>)^20, 21^.

The foregoing considerations from existing literature motivated us to work on the following fundamental aims of a broad and general interest to a wide range of FCC metals and alloys. First, confirm experimentally whether or not CTB sliding (CTBS) can actually occur in nano-twinned FCC materials. If so, establish theoretically the critical conditions under which CTBS can occur. Finally, experimentally validate the theoretical predictions in a quantitative manner with the occurrence of CTBS determined in real time and with high resolution during mechanical deformation of an FCC single crystal.

For both CTB migration (CTBM) and CTBS, the underlying mechanistic process entails the nucleation and motion of partial dislocations. The key parameter influencing this process is the resolved shear stress that is required for activating the Shockley partial dislocations. Given plasticity anisotropy (i.e., orientation-dependence) and the role of non-Schmid stress components in influencing the response of CTBs, a possible means of testing orientation-dependence of plasticity is to conduct uniaxial experiments.

Here multiple (independent) loading orientations, with respect to the orientation of CTBs, are tested whereby multiple relevant stress states involving independent combinations of pure shear (resolved shear stress) and non-Schmid (e.g., out-of-plane) shear stress/normal stress components can be imposed on the specimens in a controlled manner. We show quantitatively that CTBS is possible at least for specific loading orientations and develop a general orientation map for CTBM and CTBS. By recourse to in situ quantitative mechanical testing on nano-twinned copper pillars inside a transmission electron microscope (TEM), we demonstrate that CTBs can slide, validating our theoretical analysis and consistent with our quantitative predictions. In addition, plastic deformation is accompanied by crystal lattice rotation and re-orientation, which can eventually activate initially inactive deformation modes (such as CTBS) due to the changing Schmid factors at larger strains. We have carefully considered the changes in sample cross-section and CTB re-orientation (due to deformation induced rotation) for obtaining estimated resolve shear stresses at each CTB throughout the present study, with experimental observations consistent with the theoretical CTBM and CTBS predictions.

## Results

### Schmid factor analysis of CTBM versus CTBS

**Fig. 1:**
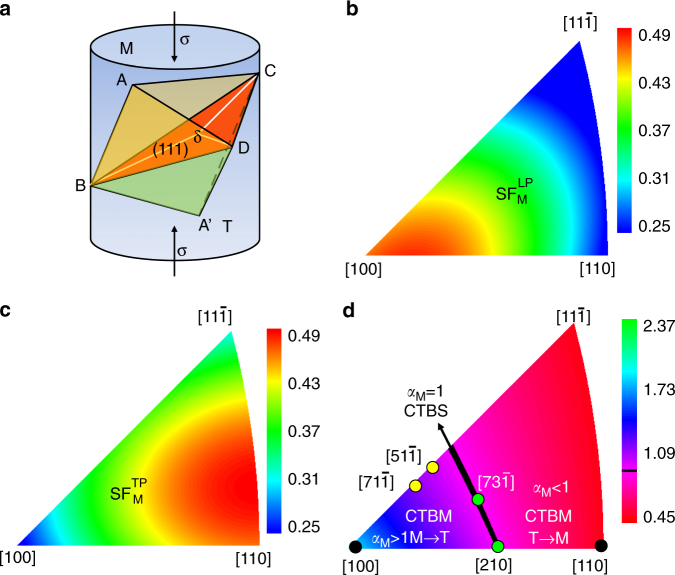
Coherent twin boundary sliding vs. coherent twin boundary migration. **a** Schematic illustration showing double Thompson tetrahedra (tetrahedron of matrix and twin) in a Cu pillar containing (111) twin planes. Color contours showing calculated values of Schmid factor of the **b** leading partial dislocation and **c** trailing partial dislocation for all the loading orientations in the $[100] - [110] - [11\overline 1 ]$stereographic triangle. **d** The standard triangle of the stereographic projection showing different regimes for coherent twin boundary sliding (CTBS) and coherent twin boundary migration (CTBM)

We begin with a consideration of the competition between CTBS and CTBM in an FCC metal, by recourse to a Schmid factor analysis. The analysis seeks to identify the most favorable crystallographic planes and directions for dislocation motion. It is widely recognized that while CTBs do not contain intrinsic dislocations, dislocations can be nucleated on free surfaces or grain boundaries where CTBs terminate and injected into the crystal to slip on the CTBs^8, 12, 14–17^. Indeed, it is the action of twinning partial dislocations that sustains CTBM^22, 23^. Specifically, CTBM is accomplished by the nucleation, and the ensuing propagation, of partial dislocations with the same 1/6<112> Burgers vector on consecutive planes (the migrating CTB). But other processes involving alternative dislocation combinations may also be possible, including those that result in CTBS without CTBM. To examine the various possibilities, we analyze the uniaxial compression deformation of a Cu crystal in Fig. 1a, in which the upper part of the specimen is designated as the matrix (M) and the lower part as the twin (T). M and T are joined at the (111) CTB, which is the plane shared by their respective Thompson tetrahedron (Fig. 1a) where dislocation slip occurs. In this work, we define ${\rm{SF}}_{\rm{M}}^{{\rm{LP}}}$and ${\rm{SF}}_{\rm{M}}^{{\rm{TP}}}$as the Schmid factors of the leading partial (LP) and trailing partial (TP) dislocations in M, respectively. Along with the change of the compression orientation, i.e., as depicted in possible changes within the $[100] - [110] - [11\overline 1 ]$ stereographic triangle, the calculated values of ${\rm{SF}}_{\rm{M}}^{{\rm{LP}}}$ and ${\rm{SF}}_{\rm{M}}^{{\rm{TP}}}$ are shown in the color contour map of Fig. 1b and Fig. 1c, respectively. Defining ${\alpha _{\rm{M}}} \equiv {\rm{SF}}_{\rm{M}}^{{\rm{LP}}}{\rm{/SF}}_{\rm{M}}^{{\rm{TP}}}$, the preference for TB migration direction can be predicted by calculating the value of *α*_M_. For example, when *α*_M_ > 1, the leading partial is always favored and the matrix part is expected to be transformed to its twin orientation, i.e., M → T, by the successive nucleation and gliding of the leading partial dislocation only. This leads to twin thickening as the CTB migrates to consume the matrix. The loading orientations corresponding to this scenario are in the blue colored region in Fig. 1d. For *α*_M_ < 1, CTB migrates into the twin part, T → M, and the loading orientations that correspond to this scenario are represented by the red colored region in Fig. 1d.

Note that there does exist a special region where *α*_M_ = 1, as marked by the black line in Fig. 1d. The Schmid factor is the same for the leading partial and the trailing partial. In this case, the leading partial dislocation and the trailing partial dislocation have the same probability of nucleation. They may alternate, that is, a leading partial can potentially be followed by a trailing partial, thereby leading to the same consequence of a perfect dislocation with a Burgers vector, **b**_full_. The leading partial increases (decreases) the twin thickness by one atomic spacing while the trailing partial decreases (increases) the twin thickness by one atomic layer. Overall, the twin thickness does not change but the crystals on either side of the CTB are displaced by **b**_full_ relative to each other. The repetition of this process (e.g., *n* times) then leads to significant CTB sliding, with a large offset ( = *n* **b**_full_) created on the surface. In other words, our analysis indicates that CTBS can exist theoretically, even though the precondition appears to be somewhat stringent.

### Experimental observations of CTBM versus CTBS

**Fig. 2:**
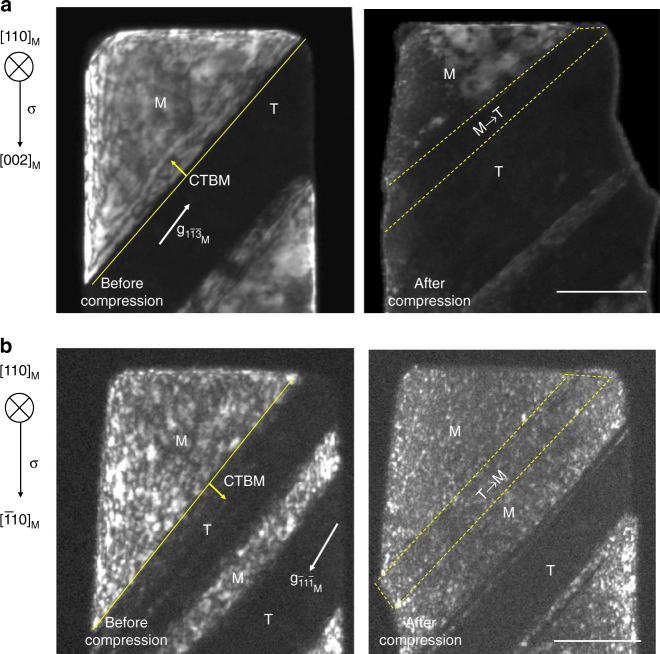
Orientation-dependent coherent twin boundary migration (CTBM). **a** [001]_M_ oriented and **b** ${[\overline 1 10]_{\rm{M}}}$ oriented Cu nanopillar. The scale bar in each figure represents 100 nm

Next, we demonstrate experimentally that not only the migration direction of the CTBs but also CTBS itself can occur in a predicable manner, through in situ compression experiments performed inside a transmission electron microscope on sub-micrometer sized Cu pillars containing nanoscale twins. We purposely fabricated pillars with different orientations from columnar-grained copper containing preferentially oriented nanoscale twins^24^ (Methods section). This facilitated uniaxial compression loading whereby all the three regions in Fig. 1d could be investigated systematically in our experiments. The loading orientations are marked by the black, green and yellow dots in Fig. 1d and listed in Table 1. The Schmid factors for the leading partial, trailing partial and the ratio *α*_M_ are also listed in Table 1. Figure 2a shows an example of CTBM toward the matrix. In this case, the copper pillar with [001]_M_ orientation was compressed in the direction shown by the left arrow, with *α*_M_ = 2.01 (Table 1). Our analysis predicts that CTBM leads to twin thickening. TEM images of the M and T regions before compression are shown in the left panel of Fig. 2a ([110] _M_ zone axis). The CTB is highlighted by the yellow solid line. As can be seen from the right panel in Fig. 2a, a part of the matrix (enclosed by yellow dashed lines) has transformed during compression to twin orientation, and consequently the entire twin region has thickened significantly. By contrast, Fig. 2b shows an example of *α*_M_ < 1, for compression of a ${[\overline 1 10]_{\rm{M}}}$ orientated sample with *α*_M_ = 0.52 (Table 1, an equivalent orientation to [110]_M_ in $[100] - [110] - [11\overline 1 ]$stereographic triangle). As predicted earlier for this case, the CTB (yellow line) migrated toward the twin part following compression. That is, a section of the twin (the region within the yellow dashed lines) was transformed into the matrix orientation. Note that the above analysis and experiment are for the uniaxial compression case. When the loading mode is changed to uniaxial tension, the CTB will migrate in the opposite direction because of the reversal of the shear stress direction (not shown). Likewise, upon tensile-compression cyclic loading, twin thickening and twin thinning would alternate^22^.

**Table 1:** Schmid factors and deformation mechanisms in nano-twinned Cu pillars

Initial pillar orientation	${\bf{SF}}_{\bf{M}}^{{\bf{LP}}}$	${\bf{SF}}_{\bf{M}}^{{\bf{TP}}}$	*α*_M_	Deformation mechanism	Number of specimens	Observation
${[100]_{\rm{M}}}$	0.47	0.24	2.01	CTBM (M → T)	2	TBM
${[110]_{\rm{M}}}$	0.25	0.48	0.52	CTBM (T → M)	2	TBM
${[210]_{\rm{M}}}$	0.42	0.42	1	CTBS	3	TBS (Supplementary Movie 1)
${[73\overline 1 ]_{\rm{M}}}$	0.43	0.43	1	CTBS	2	TBS
${[71\overline 1 ]_{\rm{M}}}$	0.45	0.32	1.40	CTBM (M → T)	3	Small strain: TBM Large strain: TBS
${[51\overline 1 ]_{\rm{M}}}$	0.44	0.35	1.25	CTBM (M → T)	2	Small strain: TBM Large strain: TBS (Supplementary Movie 4)

**Fig. 3:**
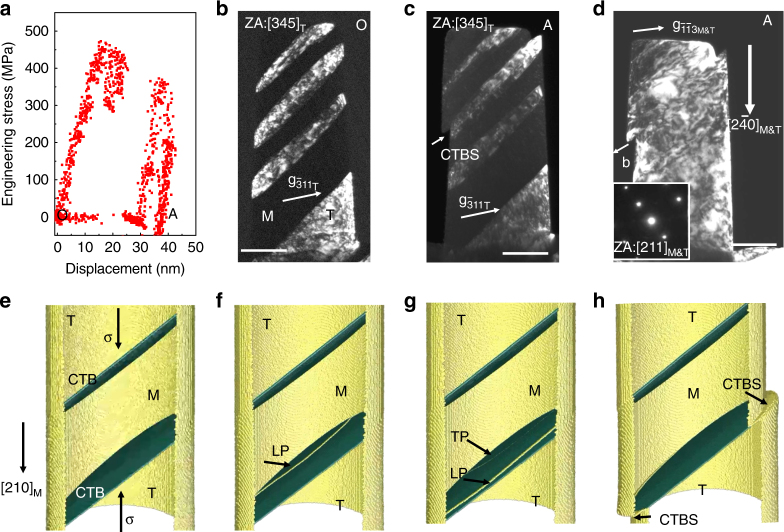
Typical example of coherent twin boundary sliding. **a** Experimentally measured mechanical response of the nano-twinned single-crystal Cu nanopillar subjected to compression along ${[1\overline 2 0]_{{\rm{M\& T}}}}$ orientation. The plot shows engineering stress as a function of loading displacement. Images **b** to **d** show dark-field TEM images of nano-twinned Cu compressed inside the TEM. Dark-field TEM image of Cu pillar **b** before and **c** after compression under [345]_T_ beam direction, and **d** after compression under [211] beam direction. The inset denotes the selected-area diffraction pattern. **e**–**h** show the evolution of coherent twin boundary sliding (CTBS) as predicted by the MD simulation. **e** A ${[210]_{\rm{M}}}$ orientated Cu pillar (an equivalent orientation to$[1\overline 2 0]$) created by MD simulation before tension. **f** A leading partial dislocation is nucleated and it propagates coherent twin boundary (CTB). **g** A trailing partial dislocation is nucleated and it propagates CTB. **h** A large surface step arising from coherent twin boundary sliding (CTBS). The scale bars in **b**, **c**, **d** represent 100 nm

Next, we consider deformation of the copper specimen for which with α_M_ ~ 1. A pillar with ${[1\overline 2 0]_{{\rm{M\& T}}}}$ orientation indexed by the selected-area diffraction pattern under [211] zone axis was compression-loaded (an equivalent orientation to [210]_M_ in $[100] - [110] - [11\overline 1 ]$stereographic triangle). The subscript ‘M&T’ here denotes that the matrix and the twin have the same lattice arrangement when they are viewed from the [211] direction. The values of ${\rm{SF}}_{\rm{M}}^{{\rm{LP}}}$ and ${\rm{SF}}_{\rm{M}}^{{\rm{TP}}}$ are both 0.42. CTBS is therefore expected, replacing CTBM. In order to increase the contrast between the twin and the matrix for clear observation of CTB deformation in the microscope, the specimen was tilted to [345] zone axis. When the uniaxial compressive stress on the specimen was about 480 MPa (Fig. 3a), CTBS was observed to initiate (Supplementary Movie 1) with a sudden and large strain burst. From comparison of the dark-field TEM images of the specimen before (Fig. 3b) and after (Fig. 3c) compression, it is evident that there is no additional shape change in the other regions of the specimen. The sliding direction is determined to be $[0\overline 1 1]$ from the diffraction pattern obtained with [211] zone axis, as seen in the inset in Fig. 3d. This indicates that the sliding is caused by a large group of 1/2<110> full dislocations that repeatedly slipped across the sample within the very short time interval corresponding to the strain burst.

### Molecular dynamics simulations of CTBS

This CTBS mechanism is further supported by our molecular dynamics simulations preformed for a [210] oriented (an equivalent orientation to$[1\overline 2 0]$) nanopillar with a diameter of 30 nm and length of ~ 58 nm (Fig. 3e; further details of the simulation can be found in the Methods). Two CTBs that are separated by 80 (111) layers were introduced into this nanopillar, which was then loaded in compression at a strain rate of 10^8^ s^−1^ at 300 K. When the uniaxial stress level reached the nucleation stress of partial dislocations, one of the CTBs exhibited sliding which was initiated by successive dislocation nucleation on the pillar surface, and followed by propagation of such dislocations. The dislocation emission was found to be in leading-trailing partial dislocation pairs. A typical CTBS sequence is shown in Fig. 3f through Fig. 3g. First, a leading partial (LP) dislocation was nucleated and it propagated on the bottom CTB (Fig. 3f). Immediately following this leading partial dislocation, a trailing partial (TP) dislocation was nucleated on the same plane and propagated across the nanopillar (Fig. 3g). While the leading partial dislocation moved the CTB one atomic layer upward, the trailing partial dislocation immediately returned it to where it was originally. The net outcome of this leading-trailing partial dislocation pair is that the CTB slides by one full dislocation Burgers vector, without causing CTBM. Sometimes, multiple leading partial and trailing partial dislocations also appear simultaneously on adjacent parallel slip planes. The detailed partial dislocation activities on CTB can be found in Supplementary Fig. 1 and viewed in Supplementary Movie 2 (see also Supplementary Note 1). Overall, the number of leading partials is approximately equal to the number of trailing partials, thus leading to significant CTBS instead of CTBM, as shown in Fig. 3h and Supplementary Movie 3. We have also carried out the simulation of the same nanopillar under tensile loading, CTBS was again observed from the morphologies change in Supplementary Fig. 2. This is expected since the equality of resolved shear stresses for leading partial and trailing partial is not affected when the loading mode is switched from compression to tension (see Supplementary Note 2 and Supplementary Fig. 3 for detailed analysis on resolved shear stress).

### Switch between CTBM and CTBS due to crystal re-orientation

As discussed earlier, a loading orientation initially favorable for CTBM may eventually become favorable for CTBS due to the rotation of the crystal with the increase in plastic strain. Correspondingly, CTBM can switch to CTBS. Two such examples are marked by the green dots in Fig. 1d. The initial crystal orientations marked ${[51\overline 1 ]_{\rm{M}}}$ and ${[71\overline 1 ]_{\rm{M}}}$ were observed to undergo CTBM first, following which they switched to CTBS after crystal rotation (see Supplementary Note 3, Supplementary Fig. 4 and Supplementary Movie 4 for one example). This suggests that the critical condition for the occurrence of CTBS as predicted by theory shown in Fig. 1 should be considered with the current configuration: the Schmid factor analysis should be predicated on the current (deformed) geometrical characteristics of the crystal and loading deformation taking into account crystal re-orientation (if there is any) due to deformation and the resultant dynamic changes to the slip system. Considering the common occurrence of crystal re-orientation during plastic deformation of ductile metals, this type of rotation-assisted CTBS would make it easier for CTBS to occur.

### Quantitative evaluation of the critical stresses for CTBS and CTBM

**Fig. 4:**
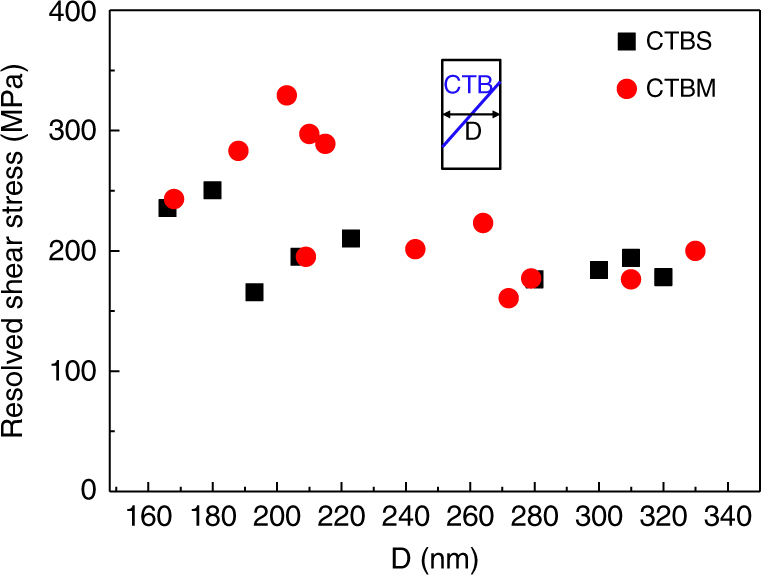
Critical resolved shear stress for coherent twin boundary sliding and coherent twin boundary migration. Experimentally determined values for the onset of coherent twin boundary sliding (CTBS) and coherent twin boundary migration (CTBM) for copper nanopillars of various diameters, D

The mechanical data accompanying our in situ TEM observation also provide an opportunity to quantify the stress at which the CTB motion occurs. In other words, the critical resolved shear stress (CRSS) for CTBS and CTBM was measured experimentally, as summarized in Fig. 4 as a function of the diameter of the pillar (at the location corresponding to the center of the CTB as indicated in the schematic in the inset of Fig. 4). Either CTBS or CTBM usually occurs with a strain burst; this is because the nucleation of the first partial dislocation is followed by the correlated nucleation^25^ of a number of other partials. The shear stress measured in our experiments can be viewed as the critical resolved shear stress needed for the nucleation event of the first 1/6<112> partial dislocation^26^. From averaging a number of measurements shown in Fig. 4, this nucleation stress is found to be 203 ± 27 MPa for CTBS and 231 ± 52 MPa for CTBM in copper.

Unlike conventional grain boundary sliding, CTBS, as observed here, does not involve either dislocation climb or diffusion. Our quantitative experimental results indicate that the resolved shear stress needed for CTBS is comparable to that for CTBM, which can be rationalized by the fact that both rely on 1/6<112> partials. This suggests that CTBS can also occur within appropriately oriented grains of polycrystalline twinned metals, possibly contributing to plastic strains along with the more commonly reported mechanism of CTBM. Excessive CTBS may result in strain compatibility problems that could instigate damage and cracking along CTB^27–29^ and could thus have consequences with respect to the mechanical integrity of heavily twinned metals^28, 30, 31^.

## Discussion

We have predicted the sliding of coherent twin boundaries in FCC crystals subjected to mechanical deformation. We have validated the predictions of the theoretical analysis quantitatively by directly observing CTBS at room temperature in twinned single-crystal copper nanopillars subjected to compression inside a TEM. The dislocations are nucleated heterogeneously from the pillar surface. A group of “short-lived interfacial dislocations” nucleate and slip across the specimen, whose correlated nucleation causes a strain burst that manifests itself as CTBS. Our analysis predicts for FCC crystals, and our experiments corroborate for the case of copper, that a special mode of boundary sliding occurs when the crystal is loaded in a particular orientation with a Schmid factor that is the same for the leading and trailing partials. Our experiments have quantitatively determined the critical resolved shear stress needed to drive CTBS, which can only happen by the nucleation of leading partial and trailing partial as a pair that undergo coordinated motion under relatively high stresses. At lower flow stresses, activities of full dislocations in the lattice would dominate the deformation in lieu of CTBM and CTBS, as shown by the example described in Supplementary Note 4, Supplementary Fig. 5 and Supplementary Movie 5. Consequently, CTBS is more likely to play a significant role for nanoscale twinned structures, since they can sustain high enough stresses to nucleate partial dislocations from the surface (in single crystals) or the grain boundary (in polycrystals). In a micro-twinned structure with length scales of twin spacing and grain size on the order of micrometers, the flow stress is generally well below the required critical stress level required for the nucleation of leading and trailing partial dislocations. CTBS is then not expected to be as likely, even if we have *α*_M_ = 1. Instead, the motion of lattice dislocations would account for plasticity. These considerations provide a rationale for the paucity of reported observations of CTBS since the vast majority of the work to date involving twins are in microcrystalline metals and alloys. While CTBM may contribute to the preservation or enhancement of ductility, CTBS could also engender stress concentrations and attendant damage and cracking along CTBs. Therefore, anisotropic twin structures^32^ might be necessary in order to balance these competing effects, so as to optimize strength, ductility and damage tolerance. The theoretical approaches and experimental findings documented in this work provide possible pathways to achieve such optimization in the design of materials microstructures.

## Methods

### Pillar fabrication

The Cu pillars were cut using focused ion beam from columnar-grained copper containing preferentially oriented nanoscale twins, which was synthesized by means of pulsed electro-deposition. A thin region of several micrometers was obtained from mechanically and chemically polishing the sample in a solution of methanol with 5 vol.% (vol/vol) nitric acid. From the thin region, pillars containing slant twin boundaries were fabricated by focused ion beam (FIB). Further details on the methods used for specimen preparation can be found elsewhere^33^. The in situ compression experiments were carried out inside a JEM 2100 field-emission-gun TEM (JEOL Ltd., Tokyo, Japan), using a Hysitron PicoIndenter (PI95) (Hysitron, Inc., Minneapolis, Minnesota, USA) under displacement control mode. The corresponding microstructure evolution was recorded using a Gatan833 (SC200) CCD camera (Gatan, Inc., Pleasanton, California, USA).

### Molecular dynamics

All simulations were performed by using the LAMMPS^34^ package and visualized by AtomEye^35^. An empirical potential developed by Mishin et al.^36^ was used to describe the interatomic interactions for Cu. The nano-twinned nanowire was created by the following procedure. First a crystal slab with matrix/twin/matrix structure was constructed by stacking (111) layers in the sequence of $ABC \cdots ABC/A/CBA \cdots CBACB/A/BCABC \cdots ABC$, where *A*, *B* and *C* denote the three typical (111) layers and twin boundaries are indicated by the slashes. Here 80 (111) layers were used for the twin part. Then the crystal slab was rotated to the desired orientation [210] and cut into a nanowire with diameter of 30 nm and length of ~ 58 nm. Periodic boundary conditions were applied along the axial direction [210]. Then the as-cut nanowire was relaxed first by energy minimization via the conjugate gradient method and then by molecular dynamics at 930 K (~ 0.7*T*_m_, where *T*_m_ is the melting temperature) under constant *NPT* (i.e., number of atoms, pressure and temperature) conditions. After the relaxation at 930 K for 100 ps, the nanowire was cooled down to 300 K within another 100 ps. Further equilibration at 300 K was performed for 20 ps and then the nanowire was subjected to compression or tensile loading. The axial loading was carried out at a constant strain rate of 10^8^ s^−1^ under constant *NVT* (*V* is the volume of simulation box) conditions. In all simulations, temperature and pressure were controlled by Nose-Hoover^37^ and Parrinello-Rhaman^38^ methods, respectively. An integration time step of 1 fs was used.

### Data availability

The data that support the findings of this study are available used to from the corresponding authors on request.

## Electronic supplementary material

Supplementary Information

Description of Additional Supplementary Files

Supplementary Movie 1

Supplementary Movie 2

Supplementary Movie 3

Supplementary Movie 4

Supplementary Movie 5
