# Supplementary material for: Sliding of coherent twin boundaries
Source: Nat Commun. 2017 Oct 24;8:1108. doi: 10.1038/s41467-017-01234-8 (PMC5715078; doi:10.1038/s41467-017-01234-8)
Supplement: Supplementary file 1 — Supplementary Information [file 41467_2017_1234_MOESM1_ESM.pdf]

Supplementary Figures

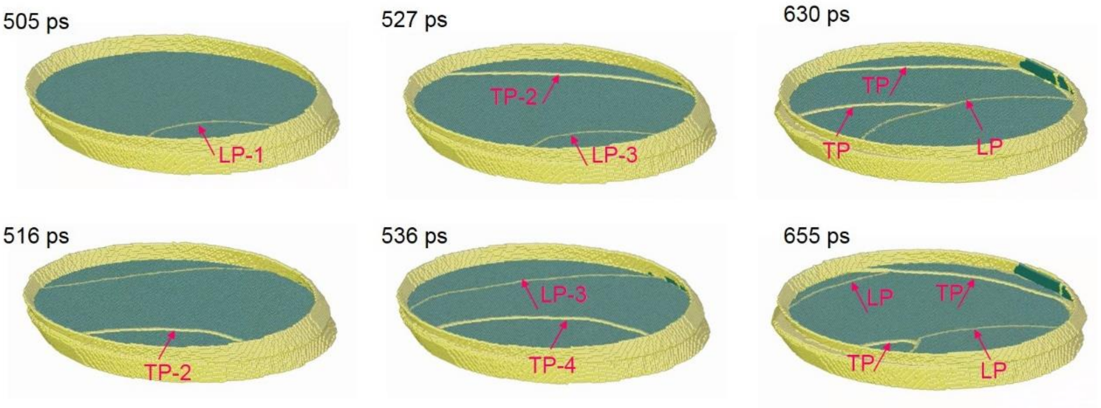

Supplementary Figure 1. Typical dislocation activities on the sliding coherent twin boundary (CTB) observed in a molecular dynamics simulation. The leading partial (LP) and trailing partial (TP) dislocations are indicated at different time points under compression.

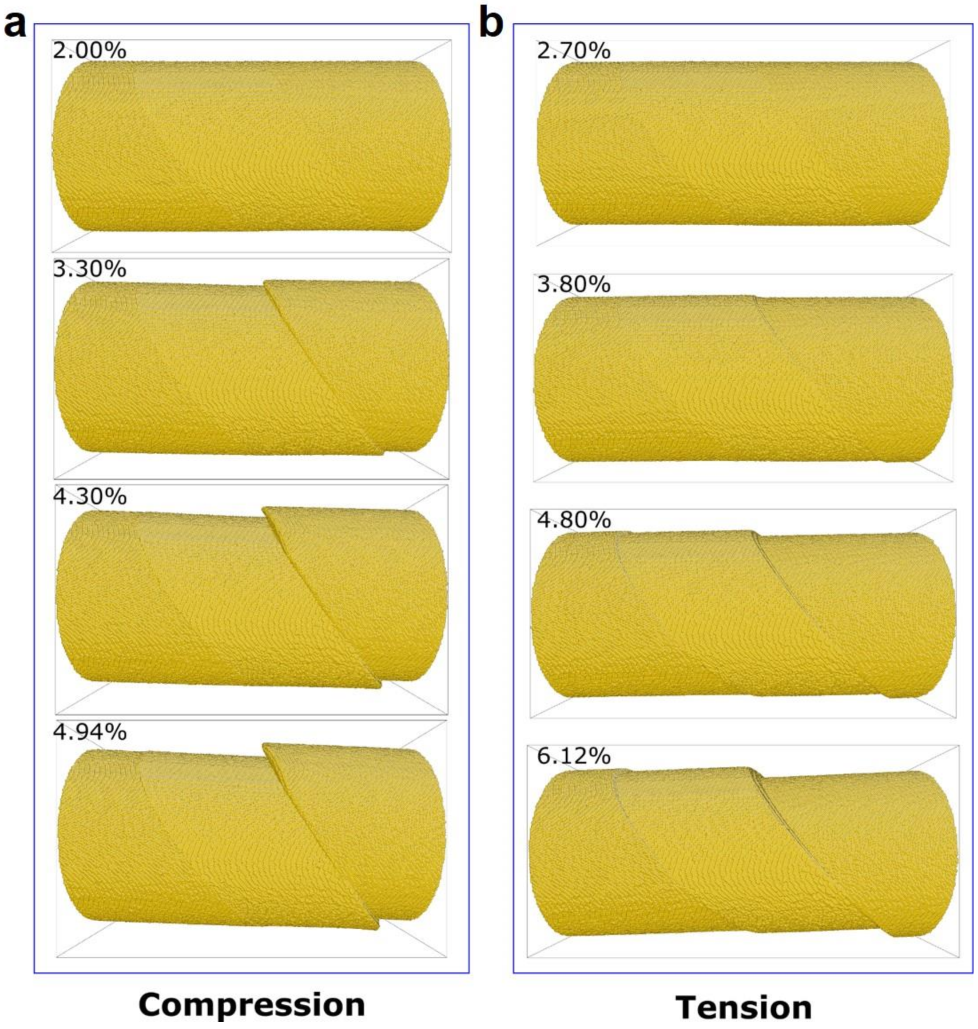

Supplementary Figure 2. Molecular dynamics simulations of a nanopillar under compression or tension. Morphology changes of a [210]-oriented Cu nanopillar at different nominal strains in molecular dynamics simulations. (a) Under compression loading. (b) Under tensile loading. Coherent twin boundary sliding (CTBS) are observed in both cases.

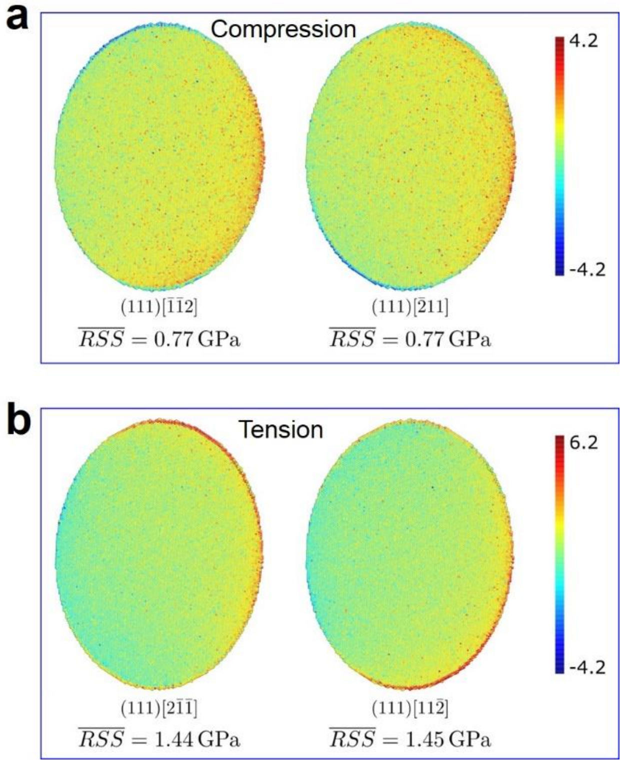

Supplementary Figure 3. Atomic resolved shear stress (RSS) distribution on a coherent twin boundary (CTB) of the nanopillar shown in Supplementary Fig. 2 right before the first partial dislocation nucleation. (a) Under compression loading. (b) Under tensile loading. The unit for the color bars is in GPa.

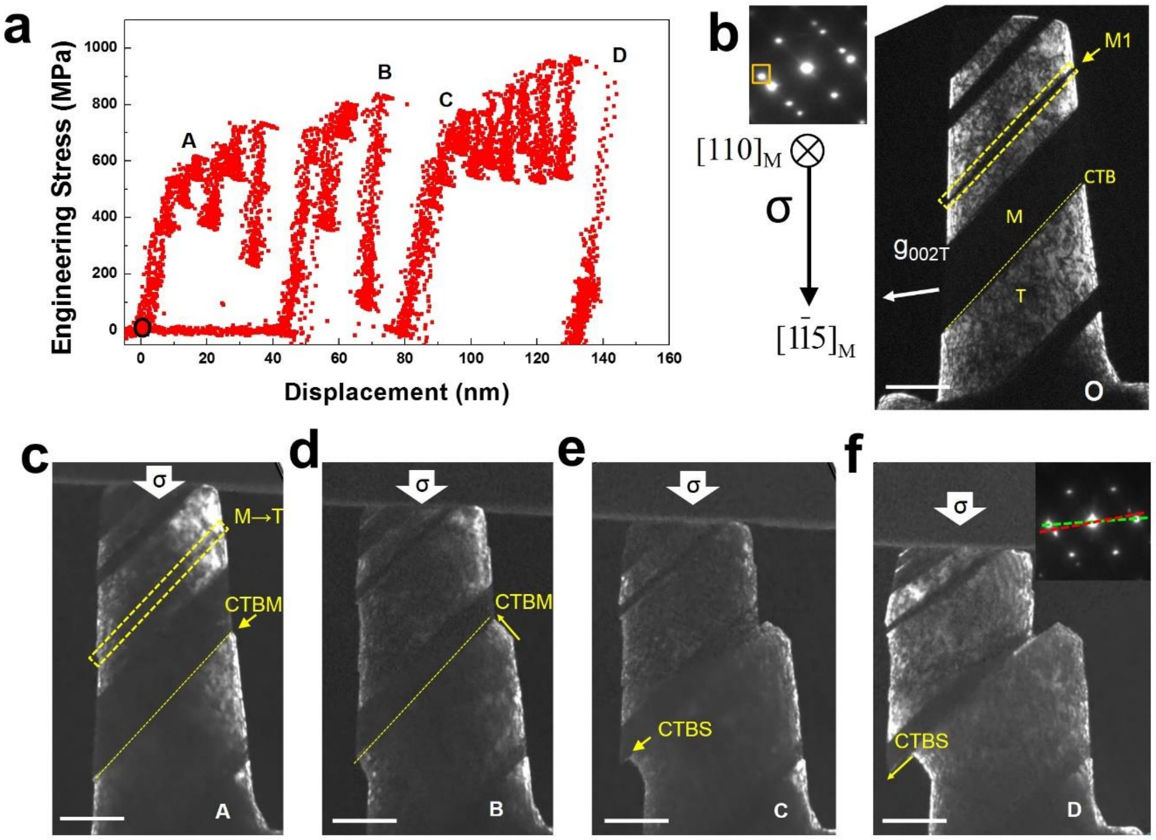

Supplementary Figure 4. A typical example showing coherent twin boundary migration (CTBM) followed by coherent twin boundary sliding (CTBS). (a) Engineering stress-displacement curve for the compression-loaded nanopillar. Bright-field TEM images of nano-twinned Cu nanopillar before compression (b), at a stress level corresponding to A (c), B (d), and C (e), and at the maximum displacement level of D (f), which are all indicated in (a). The scale bar in each sub-figure represents 100 nm.

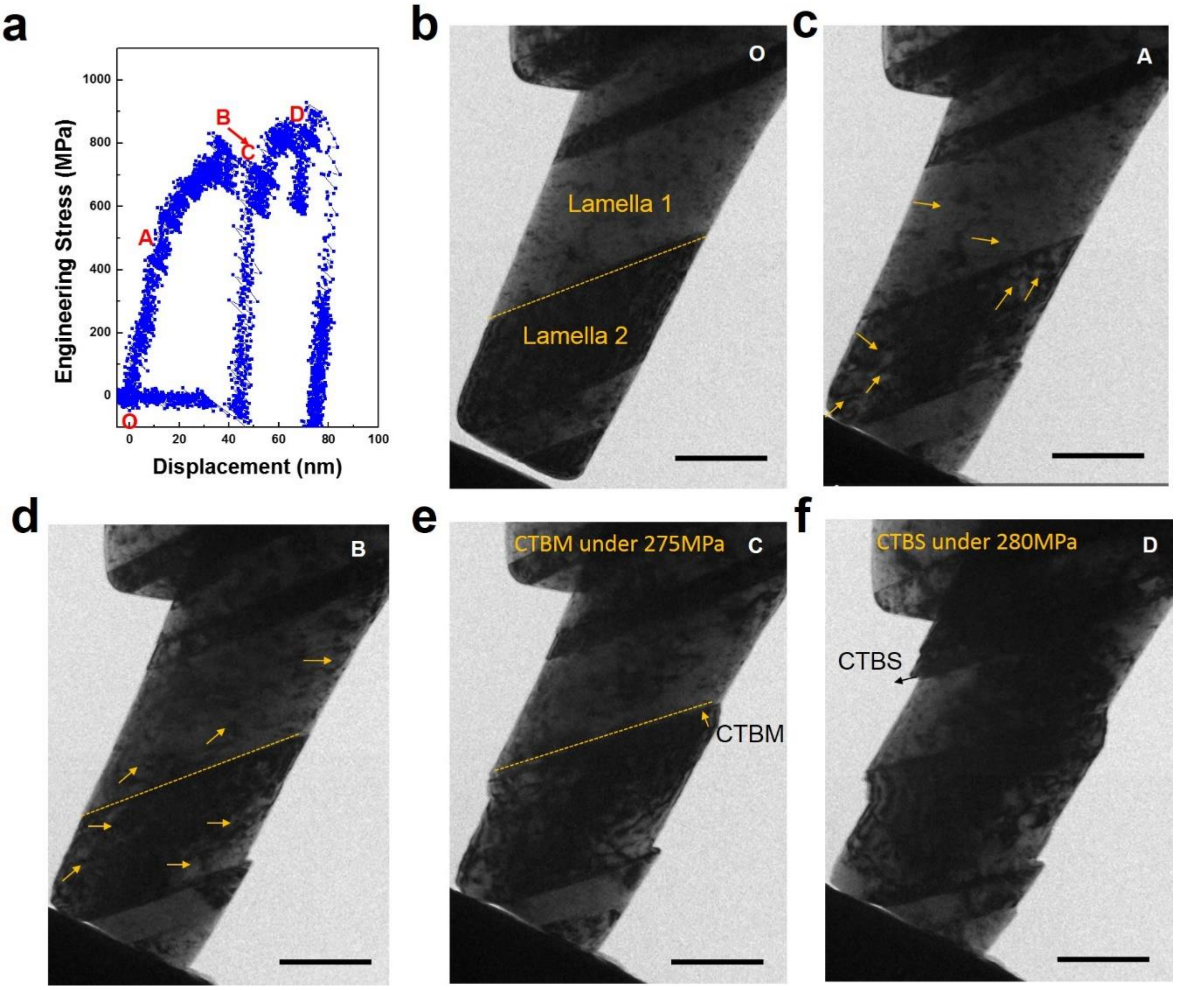

Supplementary Figure 5. Full dislocation activities before coherent twin boundary migration (CTBM) and coherent twin boundary sliding (CTBS). (a) Engineering stress-displacement curve for the compression-loaded nanopillar. (b) Bright-field TEM image of the nanopillar before compression. (c) Obvious dislocation motion at point A in (a). (d) to (e) CTBM from point B to point C in (a). (f) CTBS at point D. The scale bar in each sub-figure represents 100 nm.

Supplementary Notes

Supplementary Note 1:

Mechanism for CTBS (the repeated operation of leading-trailing partial dislocation pairs)

Generally, coherent twin boundary sliding (CTBS) was accomplished by successive nucleation and motion of leading-trailing partial dislocation pairs. Supplementary Fig. 1 shows MD simulations that illustrate the process. For example, at 505 ps, a leading partial (LP-1) is nucleated from surface and starts to glide, then a trailing partial (TP- 2) was nucleated later. As can be seen in the snapshot at 516 ps, the leading-trailing partial pair (dislocation 1-2) forms an extended dislocation which slides the CTB by one Burgers vector of a full dislocation. Such a process is repeated (see the snapshots at 527 ps and 536 ps for another subsequent leading-trailing pair dislocation 3-4) to generate more CTBS. Sometimes, multiple leading partial and trailing partial dislocations also appear simultaneously on adjacent parallel slip planes, as illustrated in the snapshots at 630 ps and 655 ps. Overall, the number of leading partials is approximately equal to the number of trailing partials, thus leading to significant CTBS instead of CTBM.

Supplementary Note 2:

Atomic RSS distribution on CTB both for compression and tension

We have measured the atomic resolved shear stress (RSS) distribution on the CTB right before the first partial dislocation nucleation for both slip systems associated with leading partial and trailing partial under compression and tension loading, respectively. The inherent structure of the configuration was first obtained by energy minimization. Then the atomic RSS was calculated by $\tau_i = (\mathbf{S}_i\mathbf{n}) \cdot \mathbf{b}$, where τ_i is the RSS of atom i, $\mathbf{S}_i$ is the stress tensor of atom i, $\mathbf{n}$ and $\mathbf{b}$ are the unit vector of slip plane normal and Burgers vector of partial dislocations. The results are shown in Supplementary Fig. 3. The average RSS for leading partial dislocation and trailing partial dislocation under compression test are both equal to 0.8 GPa, as shown in Supplementary Fig. 3a. For tension loading test, the average RSS for both slip systems are very similar: 1.44 GPa for leading partial dislocation and 1.45 GPa for trailing

partial dislocation (Supplementary Fig. 3b). This confirms that leading partial dislocation and trailing partial dislocation are equal-likely to be generated during both compression and tension, thus leading to CTBS happening during both loading modes. The difference of RSS for compression and tension is expected due to the well-known tension-compression asymmetry in homogeneous dislocation nucleation [1]. Note that, the CRSS from molecular dynamics simulation is larger than the experimentally measured value. The experimental sample is not perfect and the sample surface contains high density of defects, such as FIB induced defects, vacancies, vacancy clusters. Generally, the nucleation of partial dislocations in a defected sample has a lower nucleation stress [2]. Additionally, the very high strain rate used in molecular dynamics simulations may also contribute to the higher partial dislocation nucleation stress.

Supplementary Note 3:

CTBM and CTBS in the $[1\bar{1}5]_M$ oriented nanotwinned pillar

The loading orientations are initially favorable for CTBM in some cases. However, with the rotation of the crystal with the increase in plastic strain, it can change to a loading orientation that is favorable to CTBS, thereby facilitating a switch from CTBM to CTBS. For example, the $[1\bar{1}5]_M$ orientated nano-twinned pillar experienced CTBM first which later switched to CTBS, as shown in Fig. S4 for the $[1\bar{1}5]_M$ nanotwinned pillar (an equivalent orientation to $[51\bar{1}]_M$ in $[100]-[110]-[11\bar{1}]$ stereographic triangle). The ratio α_M was calculated to be 1.25 and the matrix was predicted to transform to twin orientation.

The as-fabricated nanotwinned structure is shown in Supplementary Fig. 4a. The twin and matrix phases are seen as light and dark regions, respectively. When the sample was compressed to a stress value indicated by point A in Supplementary Fig. 4a, the initial lamella M1 (matrix orientation) (marked by the yellow dashed rectangle in Supplementary Fig. 4b) disappeared and was transformed to the twin orientation (Supplementary Fig. 4c). At the same time, a very small surface step was found in the position marked by the yellow arrow (Supplementary Fig. 4c) due to the CTBM. Continuous CTBM resulted in a larger surface step in Supplementary Fig. 4d. Then, CTBS is observed when the stress level reached Point B in Supplementary Fig. 4a, and a surface offset along CTB was introduced by CTBS as shown by

the yellow arrow in Supplementary Fig. 4e. Subsequent deformation was dominated by CTBS and this resulted in a very large surface offset which is seen in Supplementary Fig. 4f. The diffraction pattern shown in the inset in this figure indicates that the crystal orientation was indeed rotated by about 5^0, red dashed line to green dashed line (the change of $[002]_T$ reflection vector direction).

Supplementary Note 4:

Full dislocation activities in the $[1\bar{1}5]_M$ oriented nanotwinned pillar

One example for the full lattice dislocation activities is shown in the $[1\bar{1}5]_M$ oriented nanotwinned pillar in Supplementary Fig. 5. The $[1\bar{1}5]_M$ orientated nano-twinned pillar was also observed to experience CTBM first which later switched to CTBS. Before CTBM and CTBS, obvious full lattice dislocation activities were observed. In lamella 1 and lamella 2, a high density of dislocations were pre-existed before compression (Supplementary Fig. 5b). With the increase of the compressive stress (to point A in Supplementary Fig. 5a), some of the pre-existed dislocations started to move, as shown by the snapshot of the movie (marked by the yellow arrows in Supplementary Fig. 5c). The CTBs were still stationary until the critical resolved shear stress reached the critical value of CTBM (275 MPa); the CTB marked by the yellow dashed line started to migrate from Supplementary Fig. 5d to Supplementary Fig. 5e in a strain burst from point B to point C in Supplementary Fig. 5a. With increasing plastic loading, the CTB marked by the red dashed line stated to slide when the CRSS reached the critical value for CTBS (280 MPa), as shown in Supplementary Fig. 5f. In other words, the full dislocation activities almost existed throughout the whole deformation process, while the CTBM and CTBS only happened when the flow stress reached the critical value for CTBM and CTBS.

Supplementary References

1. Tschopp MA, McDowell DL. Tension-compression asymmetry in homogeneous dislocation nucleation in single crystal copper. *Appl Phys Lett* **90**, 121916 (2007).

2. Wang Z-J, Shan Z-W, Li J, Sun J, Ma E. Pristine-to-pristine regime of plastic deformation in submicron-sized single crystal gold particles. *Acta Materialia* **60**, 1368-1377 (2012).
