# Supplementary material for: Sliding of coherent twin boundaries
Source: Nat Commun. 2017 Oct 24;8:1108. doi: 10.1038/s41467-017-01234-8 (PMC5715078; doi:10.1038/s41467-017-01234-8)
Supplement: Supplementary file 2 — Description of Additional Supplementary Files [file 41467_2017_1234_MOESM2_ESM.docx]

Description of Additional Supplementary Files

File Name: Supplementary Movie 1

Description: Coherent twin boundary sliding in a nanopillar subjected to compression along orientation.

File Name: Supplementary Movie 2

Description: Molecular dynamics simulation under compressive load, showing typical dislocation activities on the sliding coherent twin boundary.

File Name: Supplementary Movie 3

Description: Coherent twin boundary sliding in a [210] oriented nanopillar under compression, as predicted by the molecular dynamics simulation.

File Name: Supplementary Movie 4

Description: A typical example showing coherent twin boundary migration followed by coherent twin boundary sliding.

File Name: Supplementary Movie 5

Description: Full dislocation activities before coherent twin boundary migration and coherent twin boundary sliding.
